# Trace fossil evidence for infaunal moulting in a Middle Devonian non-trilobite euarthropod

**DOI:** 10.1038/s41598-020-62019-6

**Published:** 2020-03-24

**Authors:** M. Gabriela Mángano, Javier Ortega-Hernández, Laura Piñuela, Luis A. Buatois, Francisco J. Rodríguez-Tovar, José Carlos García-Ramos

**Affiliations:** 1 Department of Geological Sciences, University of Saskatchewan, 114 Science Place, Saskatoon, SK S7N 5E2 Canada; 2 Museum of Comparative Zoology and Department of Organismic and Evolutionary Biology, Harvard University, 26 Oxford Street, Cambridge, MA 02138 USA; 3 Museo del Jurásico de Asturias (MUJA), 33328 Colunga, Asturias Spain; 4 Departamento de Estratigrafía y Paleontología, Universidad de Granada, 18002 Granada, Spain

**Keywords:** Palaeontology, Palaeoecology

## Abstract

Trace fossils represent the primary source of information on the evolution of animal behaviour through deep time, and provide exceptional insights into complex life strategies that would be otherwise impossible to infer from the study of body parts alone. Here, we describe unusual trace fossils found in marginal-marine, storm- and river-flood deposits from the Middle Devonian Naranco Formation of Asturias (northern Spain) that constitute the first evidence for infaunal moulting in a non-trilobite euarthropod. The trace fossils are preserved in convex hyporelief, and include two main morphological variants that reflect a behavioural continuum. Morphotype 1 consists of a structure that superficially resembles a *Rusophycus* with an oval outline that possesses a distinctly three lobed axis with an elevated central ridge and regularly spaced transverse furrows that convey the appearance of discrete body segments. The anterior part is the most irregular region of the structure, and it is not always recorded. Morphotype 2 displays more elongated, tubular morphology. Careful observation, however, reveals that it comprises up to three successive morphotype 1 specimens organised in a linear fashion and partially truncating each other. Trilobate morphology and effaced transverse furrows are locally evident, but the predominant morphological feature is the continuous, elevated ridge. The detailed morphology of morphotype 1 and well-preserved, discrete segments of morphotype 2 closely resemble the dorsal exoskeleton of the enigmatic late Carboniferous euarthropod *Camptophyllia*, suggesting the possible affinities of the producer. Comparisons with patterns of Devonian phacopid trilobite exuviation suggest that the Naranco Formation trace fossils may have been produced by the infaunal activities of an euarthropod that anchored its dorsal exoskeleton in the firm sediment during the body inversion moult procedure. Our findings expand the phylogenetic and environmental occurrence of infaunal moulting in Palaeozoic euarthropods, and suggest a defensive strategy against predation, previously only known from trilobites preserved in open-marine deposits.

## Introduction

The process of ecdysis – in which the exoskeleton is discarded and substituted with a new one in order to accommodate changes in size and/or shape during growth – represents a fundamental biological constraint that has shaped the evolution of euarthropods for more than half a billion years^1,2^. The abundance of euarthropod body fossils in the rock record has led to a comprehensive understanding of the morphological adaptations required for ecdysis, namely the distribution and variability of suture lines on the dorsal exoskeleton of major groups including trilobites and eurypterids^1,3–5^. These investigations have also produced insights on euarthropod palaeoecology through deep time, such as environmental triggers for mass moulting events^4,6,7^, adaptive advantages of particular moulting strategies^5^, and behavioural tradeoffs in response to predation^8,9^. However, the fossil record of moulting is strongly biased in favour of biomineralizing groups inhabiting open-marine environments, particularly during the Palaeozoic^1^, and thus the current understanding of this critical aspect of euarthropod evolution is drastically underrepresented for soft-bodied organisms.

Here, we describe unusual trace fossils from the Middle Devonian Naranco Formation of northern Spain^10^ that provide evidence of shallow infaunal moulting in an euarthropod taking place in a marginal-marine depositional environment. Our findings represent one of the earliest unequivocal trace fossils produced by moulting (ecdysichnia)^11^, and expand the record of infaunal moulting to include non-trilobite euarthropods^9^. The infaunal strategy revealed by these peculiar trace fossils may reflect a response to predation pressure on the behaviour of organisms inhabiting shallow- to marginal-marine environments during the mid-Palaeozoic^8^.

## Geological and sedimentological context

**Figure 1:**
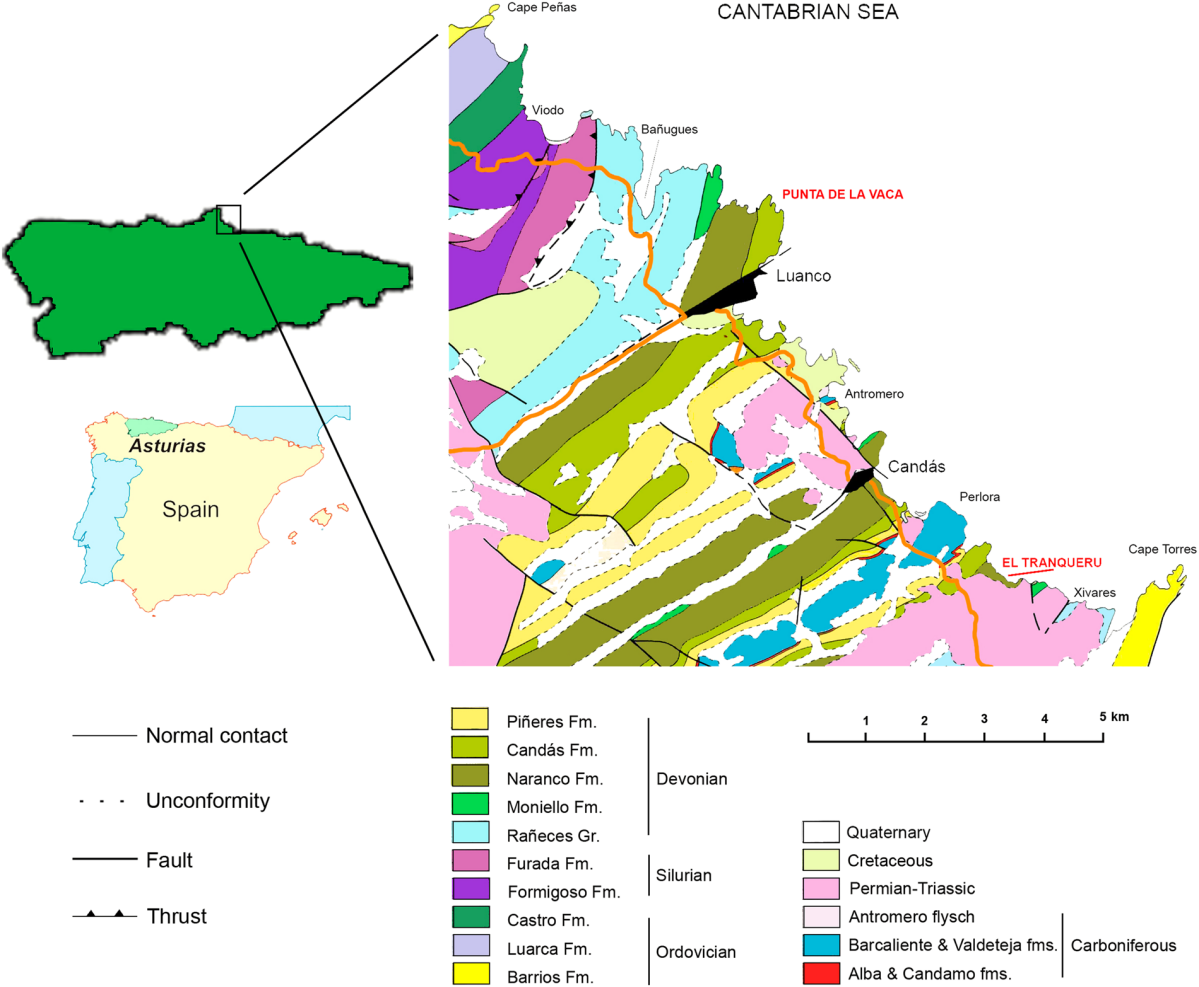
Location and geological map of the area between Cabo Peñas and Cabo Torres in Asturias, northern Spain. Modified from ref. ^24^. Maps redrawn by Laura Piñuela using CorelDraw software version 12.

The studied material comes from the Middle Devonian (Eifelian-earliest Givetian^12^) Naranco Formation of Asturias in northern Spain (Fig. 1), which mainly consists of intercalations of sandstone and mudstone, with several oolitic ironstone beds^10^. This unit accumulated in a wave-influenced, marginal- and shallow-marine environment that was affected by fluvial discharge during storm floods.

**Figure 2:**
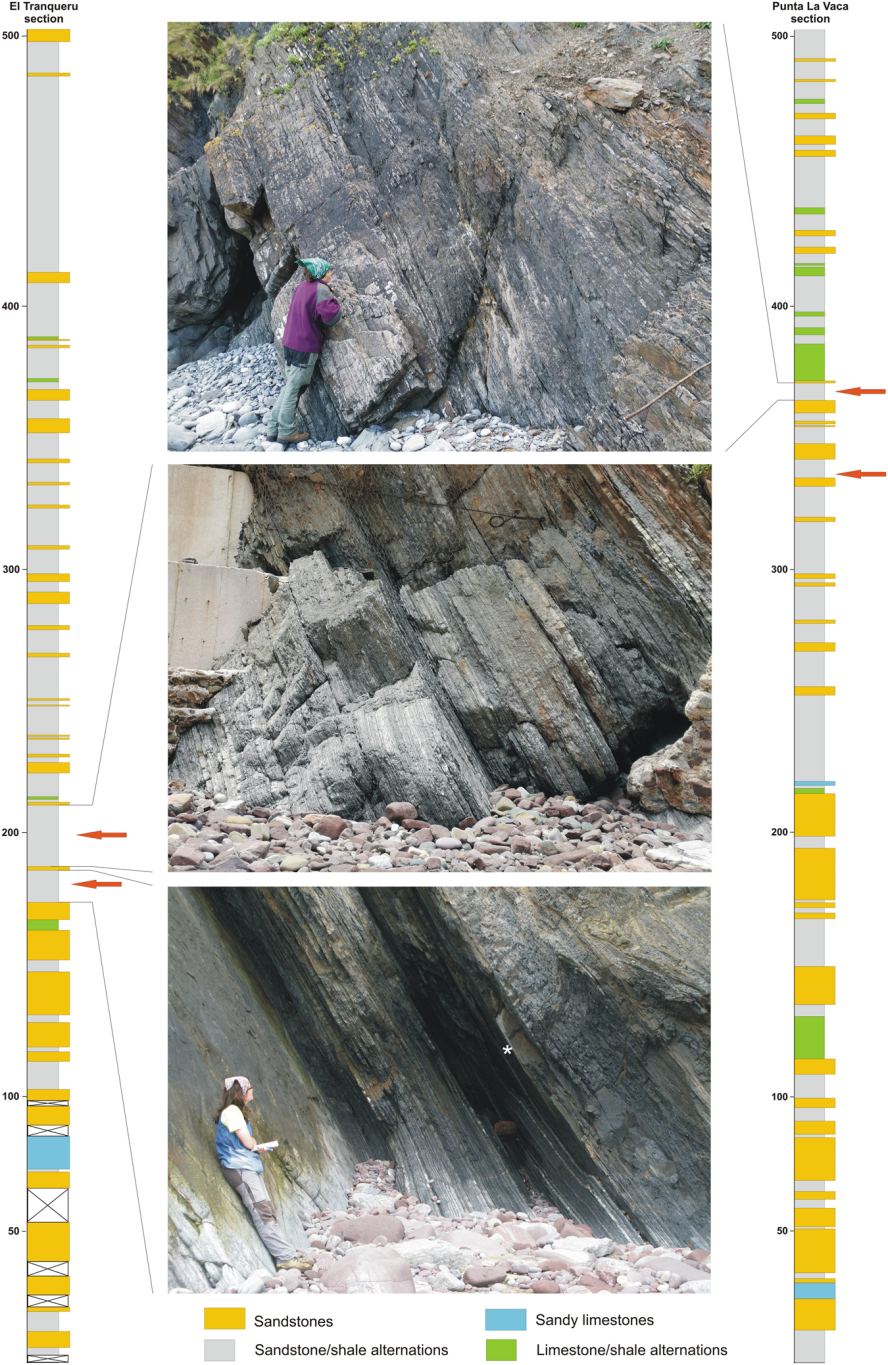
Synthetic sedimentological logs of El Tranqueru and Punta La Vaca sections, showing the stratigraphical position of the intervals containing the studied trace fossils, as well as representative photos of the ichnofossil-bearing deposits. Logs drawn by Laura Piñuela using CorelDraw software version 12.

The new trace fossils were found in two coeval sections of similar thickness (approximately 500 m): El Tranqueru and Punta La Vaca (Fig. 2). Both localities are 7 km apart and occur along the coastal cliffs of the Cantabrian Sea. These two sections represent some of the best-preserved and thickest siliciclastic successions of Middle Devonian rocks within the Cantabrian Zone, recording a complete succession of the whole Naranco Formation. The studied trace fossils occur in two intervals in each of the sections. However, additional loose specimens were found, indicating that these trace fossils are present in similar facies intervals through the succession in both localities.

In particular, the trace fossils documented in this study occur at the bases of 0.3–5.0 cm thick, erosionally based, very fine- to fine-grained sandstone locally showing parallel lamination, and starved, linguoid and combined flow ripples. Sole marks (e.g. groove casts) are common. These sandstone beds are typically separated by mudstone layers. Some sandstone beds and mudstone intervals display soft-sediment deformation structures, such as convolute lamination, load casts, and slumps of various scales. Overall these deposits are sparsely bioturbated. Interbedded, relatively thick and normally graded mudstone units tend to be unbioturbated.

Characteristic ichnotaxa commonly associated to the studied biogenic structures include *Conostichus*, *Planolites* and *Teichichnus*, among others^13,14^. Ichnodiversity is low to moderate, reflecting the depauperate or stressed *Cruziana* Ichnofacies. The low ichnodiversity and the sparse bioturbation, coupled with the sedimentologic evidence, suggest various stressors, such as freshwater discharge, high rates of sedimentation, and erosion and by-pass. The ichnofossil-bearing intervals are interpreted as representing river- and storm-flood deposits, characterised by the interplay of hyperpycnal flows and wave action.

## Results

**Figure 3:**
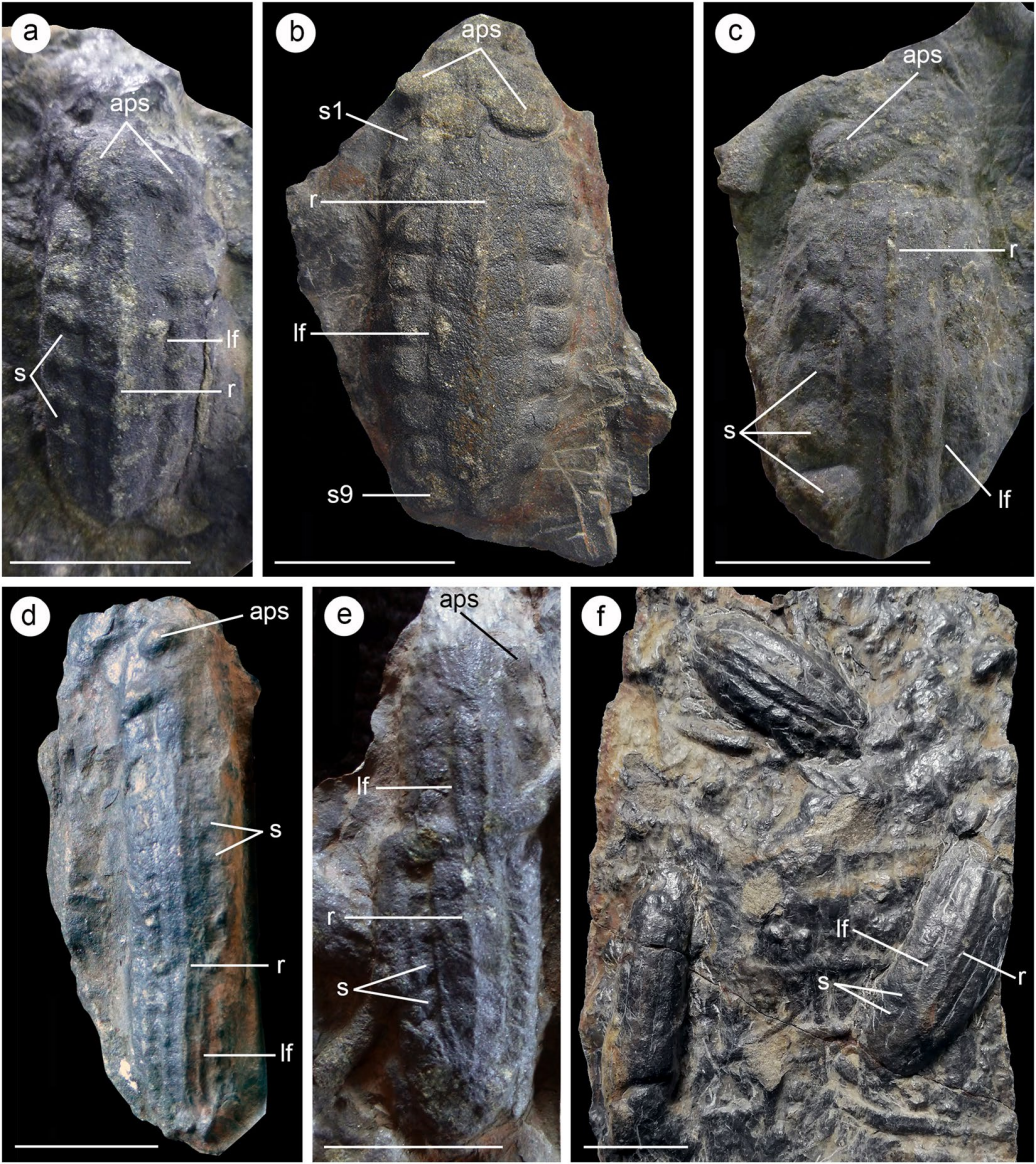
Infaunal moulting trace fossils from the Middle Devonian Naranco Formation in Asturias, northern Spain. (**a**) Morphotype 1, MUJA-4061; (**b**) Morphotype 1, MUJA-4662; (**c**) Morphotype 1, MUJA-4002; (**d**) Morphotype 2, MUJA-4660; (**e**) Morphotype 2, MUJA-4661; (**f**) Morphotype 1 (upper specimen) and Morphotype 2 (lower specimens), MUJA-4814. Abbreviations: r, central ridge; lf, longitudinal furrows; s1-9, body segments; aps, anterior paired structures. Scale bars are 2 cm.

Two different morphotypes have been identified based on the study of 65 collected specimens and 33 additional ones studied in the field. Of the ones collected, 46 correspond to morphotype 1 and 19 to morphotype 2. The new ichnofossils from the Naranco Formation consist of bilaterally symmetrical structures with clear antero-posterior orientation, and a distinctive trilobate morphology preserved in convex hyporelief (Fig. 3a–f).

**Figure 4:**
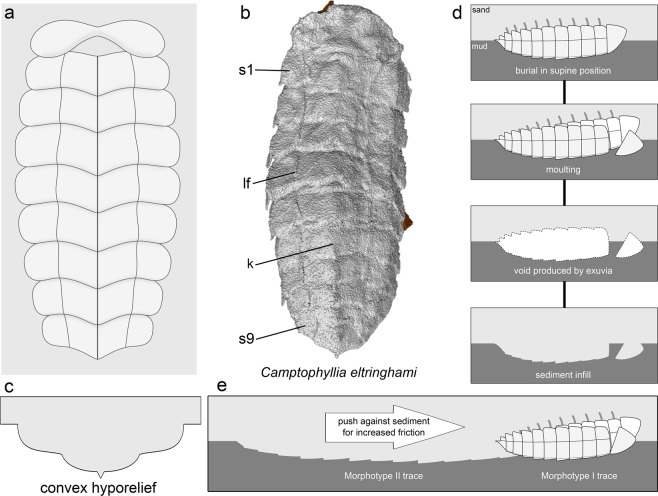
Taphonomy of moulting trace fossils from the Naranco Formation. **(a)** Reconstruction of morphotype 1 trace fossil. **(b)** Tomographic reconstruction of *Camptophyllia* dorsal exoskeleton [after Ref. ^16^]. **(c)** Transverse section of morphotype 1 trace fossil in convex hyporelief resulting from sediment infill. **(d)** Reconstructed sequence of body inversion moult procedure resulting in morphotype 1 trace fossils produced by a *Camptophyllia*-like animal. **(e)** Reconstruction of trace fossil formation resulting in morphotype 2. Abbreviations as in Fig. 3. Reconstructions drawn by Javier Ortega-Hernández using Inkscape.

Morphotype 1 consists of convex trilobated structures with an oval outline characterised by the presence of an axial region marked by two longitudinal furrows that define three lobes along the length of the trace fossil, the axial of which is wider and deeper than the lateral ones. Lobes display regularly spaced transverse furrows that evoke the appearance of body segments that gently taper in width posteriorly (Figs. 3a–c and 4a). Best-preserved specimens record up to nine discrete segments and an anterior area with convex subcircular paired structures (Fig. 3a,b). Within each of the segments, the axial lobe has an approximately subpentagonal shape with a posteriorly facing apex, and also features an elevated central ridge (Figs. 3a–c and 4a). By contrast, the segments that form the lateral lobes have a subrectangular outline with rounded distal margins. Morphotype 1 displays a range of variability in the preservation of morphological features (Figs. 3a–c and S1a–e). Poorly preserved specimens typically display mostly smooth surface with an oval outline, subtle trilobation, and an anterior area characterised by either the convex subcircular, paired structures (Fig. S1a) or a fan-like convex protuberance (Fig. S1b–d). The axial midline ridge is present in the vast majority of specimens (Figs. 3a–c and S1b,c), only being absent in some poorly preserved variants (Fig. S1d,e). The trace fossils belonging to morphotype 1 are 19–56 mm long (mean of complete specimens = 40 mm) and 11–23 mm wide (mean of complete specimens = 18 mm) (Tables S1 and S3). Average (mean) length considering the totality of morphotype 1 specimens (including specimens partially covered by mudstone or intercepted by a trace fossil, which complicates measurements) is 39 mm (Tables S1 and S3).

Morphotype 2 is similar to morphotype 1 in overall appearance, including the presence of a well-defined axial lobe with an elevated, central ridge, but differs in having a much more elongate shape, defining almost a tubular structure (Figs. 3d–f and S2a). The best-preserved specimens of morphotype 2 show the presence of transverse regular furrows that convey a segmented appearance, but these are significantly effaced compared to those expressed in morphotype 1 (Fig. 3d,e). Under close inspection, some specimens of morphotype 2 show two to three consecutive, partially truncated morphotype 1 specimens forming a linear structure (Fig. S2a–c). This is typically revealed by subtle interruptions in the curve profile as observed in cross-sectional view (Fig. S2b,c). Some preservational variants of morphotype 2 are smooth, lacking any transverse segmentation (Fig. 3f, lower left). However, close examination reveals subtle longitudinal furrows (i.e. trilobate morphology) and/or the distinctive, central ridge. The subcircular anterior paired structures or fan-like protuberances are only rarely preserved at the anterior end of some morphotype 2 specimens (Fig. 3d). The trace fossils belonging to morphotype 2 are 31–115 mm long (mean = 63 mm) and 7–24 mm wide (mean = 16 mm) (Tables S2 and S3).

Density of trace fossils preserved on the bases of sandstone beds may be locally high. Detailed analysis of a superbly exposed bedding plane containing 33 specimens suggests densities of between 8 and 14 specimens per m^2^ (Figs. S3 and S4). Study of collected rock slabs may suggest that specimens are apparently oriented in random directions (Fig. 3f). However, specimens in the sandstone surface previously mentioned clearly display a preferential orientation (Az 34.7°) (Fig. S3).

## Discussion

### Mode of formation

The overall morphology and variability in the preservation of the Naranco Formation trace fossils analysed here are best regarded as the result of different facets of a single anatomy defining a behavioural continuum. In this context, the almost pristine body impression with transverse furrows defining the segments observed in best-preserved specimens of morphotype 1 (Fig. 3a–c) can be related to the effacement of these features in morphotype 2 (Fig. 3d–f). This suggests that these differences correspond to morphotype 1 documenting momentary pause (i.e. stasis) and elongated morphotype 2 recording significant translational movement through the sediment. However, the fact that the axial lobe and central elevated ridge are consistently expressed in both morphotypes indicates that, although morphotype 1 records for the most part a stationary structure producing an impression that mimics the dorsal anatomy of the tracemaker, there was a subtle displacement involved in its generation (contra body fossil interpretation). This is revealed by the continuous nature of the midline ridge, most likely recording discrete spines/keel in the exoskeleton of the producer (cf. Figs. 3b and 4b). The variability of the anterior part of morphotype 1, locally with a fan-like mounded morphology (S1b–d), indicates disturbance of the sediment related to animal-sediment interaction, therefore supporting a trace fossil origin. In fact, there is a real gradation between morphotypes 1 and 2, reflecting a morphological and behavioral continuum as clearly documented by some long specimens of morphotype 2 (Figs. 3d,e and S2). This evokes similarities with intergrading *Rusophycus* and *Cruziana*, which are widely regarded as biogenic structures recording resting/stationary (*Rusophycus*) and combined locomotion and feeding activity (*Cruziana*) of benthic deposit feeding euarthropods with homonymous limbs^15^. Morphotype 1 implies the docking of the dorsal part of the exoskeleton in the firm mud, producing an oval symmetrical structure superficially resembling *Rusophycus*, whereas morphotype 2 involves concatenated morphotype 1 specimens resulting in an elongated structure recording linear movement through the sediment akin to *Cruziana* (Fig. 4e). In particular, the concatenation of truncated morphotype 1 specimens can be compared with the gradation between *R*. *eutendorfensis* and the resultant *C*. *tenella* generated by repeated, partially overlapping *R*. *eutendorfensis* segments^16^.

Comparisons with some ichnospecies of *Rusophycus* and *Cruziana* refer only to the stop-start mechanism resulting in concatenated static elements (i.e. *Rusophycus*-like segments) to create linear structures recording locomotion (*Cruziana*-like). Contrary to *Rusophycus* and *Cruziana*, however, the Naranco Formation trace fossils are not the result of the interaction of walking legs or ventral anatomy against the sediment; they lack leg striations (i.e. “scratch imprints”) or any kind of appendage impressions (e.g. coxal impressions, exopodite brushings) found in legitimate euarthropod trackways and burrows. Instead, these trace fossils are best interpreted as the product of the dorsal exoskeleton of an euarthropod pressed infaunally against the cohesive muddy sediment. This resulted in the distinctive trilobate morphology with a deeper axial lobe and segmented appearance expressed in morphotype 1, and the effaced surface of elongated morphotype 2 with the axial ridge. Initial anchoring of the dorsal exoskeleton (morphotype 1) was occasionally followed by subsequent dragging and re-anchoring in an adjacent consecutive position generating partially overlapping replicas of the dorsal anatomy in a continuous structure (morphotype 2). Successive animal body re-adjustments are recorded by subtle angle changes defining nested curve segments in morphotype-2 cross-sectional profile (Fig. S2b,c). In this context, it is worth drawing attention to the moulting behavior of some extant chelicerates (e.g. arachnids), in which ecdysis is performed in a supine position inside a burrow, and in which the emerging individual escapes the exuvium through an aperture of the anterior exoskeletal margins^4^.

### The tracemaker and its environmental preference

Attribution of a trace fossil to a particular producer is exceptional in the ichnological record. There is a genuine interest in palaeobiology, however, in deciphering tracemakers. Linking these two records could unravel previously inaccessible ecological and evolutionary information. A trilobite producer is unlikely, as the detailed preservation of the Naranco Formation trace fossils would allow the identification of diagnostic morphological features, such as the axial ring ornamentation and pleural furrows^1,3,8,9^. Although the identification of the producer of the Naranco Formation trace fossils poses a significant challenge, the exquisite preservation of some specimens of morphotype 1 allows for direct comparison with the enigmatic non-trilobite euarthropod *Camptophyllia eltringhami* from the upper Carboniferous (i.e. Pennsylvanian) British Coal Measures^17,18^. In fact, the enigmatic Naranco trace fossils closely resemble the dorsal trunk exoskeleton of *Camptophyllia* in overall shape, size range, the presence of paired longitudinal furrows forming three lobes, wide axial lobe with an elevated central keel, the presence of nine segments that taper in width posteriorly, and even a pointed back end (Fig. 4a,b), strongly supporting that the producer was in all likelihood a *Camptophyllia*-like, benthic euarthropod. The Naranco Formation trace fossils differ only from the exoskeleton of *Camptophyllia* in the absence of pleural spines and the shape of the anterior region, which display wide variability in the sedimentary biogenic structure, suggesting anatomical disturbance of this region during animal-substrate interaction resulting in emergence. Despite the close morphological similarities between the Naranco Formation trace fossils and the dorsal exoskeleton of *Camptophyllia*^18^, it is difficult to assess whether this euarthropod and the trace maker were phylogenetically closely related, or simply the result of convergent evolution, given the lack of ventral anatomical information in both cases. The higher affinities of *Camptophyllia* are a source of controversy all by themselves, as its exoskeleton has prompted comparisons with oniscid isopod crustaceans, arthropleurid myriapods, and even euthycarcinoids^18^. Given this uncertainty, we argue for a conservative interpretation of the Naranco Formation trace fossils as being produced by a *Camptophyllia*-like euarthropod with a dorsal exoskeleton that closely resembles that of a oniscid isopod based only on their similar dorsal morphology.

*Camptophyllia* and walking traces tentatively attributed to it are present in late Carboniferous delta-plain lacustrine settings characterised by freshwater conditions^17,18^. In contrast, the Devonian trace fossils documented in this study are present in deposits that, although inferred to have been formed in connection with a river-mouth, record more distal settings where normal marine salinities were repeatedly affected by freshwater discharge, resulting in periods of brackish-water conditions. This palaeoenvironmental discrepancy could in principle be explained in two different ways. First, the producers may have originally lived in proximal delta-plain settings (as observed in *Camptophyllia*), but been entrained in the hyperpycnal flows and transported seaward (as observed in the Naranco Formation). Second, the producers of the Naranco trace fossils may have actually lived in these shallow marginal-marine settings. If this is the case, these *Camptophyllia*-like euarthropods with a similar functional morphology (and likely ecology) to that of oniscid isopods could have originated in fully marine environments during the Devonian and subsequently migrated landwards during the Carboniferous. These environmental shifts through time are not unusual, and have been detected in various arthropod groups^19^, including those taxa more directly comparable with *Camptophyllia* (i.e. oniscid isopod, arthropleurids, euthycarcinoids)^18^. Further exploration of Devonian and Carboniferous strata may reveal similar biogenic structures to those herein described, providing crucial evidence to evaluate these competing hypotheses.

### Behavioural significance

The conclusion that the Naranco Formation trace fossils were most likely produced by *Camptophyllia*-like euarthropods living in shallow- to marginal-marine environments begs the question: what particular behaviour do these unusual fossils reflect? The convex hyporelief preservation (Fig. 4c), similarity with the dorsal exoskeleton of *Camptophyllia* (Fig. 4b), and lack of a recognizable head region indicate that the trace fossils reflect an impression of the dorsum pushed against the sediment whilst the animal was in upside down position. This configuration is consistent with the *body inversion moult procedure* envisaged for Devonian phacopid trilobites (Fig. 4e)^1,3^, which involved the active burial of the animal in an upside down position, followed by forceful thrusts of the exoskeleton against the surrounding sediment in order to facilitate the moulting process. However, the Naranco material records unusually pristine structures that seem not to involve any significant struggle in the process of ecdysis. It is worth noting that ecdysis need not to be a highly energetic strategy^1,4^. In the case of the Naranco Formation structures, the clue resides in the nature of the hosting sediment, in particular its high consistency. In addition to the richly ornamented trace fossils present in these deposits (e.g. *Conostichus*), sedimentological evidence indicates frequent erosional (e.g. groove casts) and exhumation processes providing optimal conditions for firm substrates. The preferred orientations of specimens preserved on the large surface is consistent with relatively high-energy conditions under the action of currents. In this context, morphotype 1 records an upside-down resting stance anchored in a firm mudstone, whereas morphotype 2 resulted from a more prolonged process involving successive attempts of anchoring and release of the exoskeleton. This latter process resulted in repeated, consecutive, partially overlapped structures defining a linear structure (morphotype 2) spanning several centimeters. In our interpretation, the anterior region reflects a moulting strategy involving the disarticulation of the cephalic shield (Fig. 4e). This region characterised either by an irregular, fan-like mounded protuberance or a pair of subcircular highly convex structures was strongly disturbed during emergence dislodging the remainders of the ruptured cephalic cuticle. The high density of moulting structures recorded in the surface analysed is consistent with mass moulting events, as recorded for different groups of arthropods elsewhere^6,7^.

The Naranco Formation trace fossils represent the first record of infaunal mass moulting in a soft-bodied euarthropod within a marginal-marine depositional setting, and significantly expand the known phylogenetic and environmental occurrence of this complex behaviour during the Palaeozoic. Previous trace fossil evidence for Palaeozoic moulting is based on the ichnogenus *Rusophycus* and essentially restricted to trilobites^20,21^. Cryptic behaviour in trilobites – including infaunal^8,9^ and sheltered moulting^21^ – has been regarded as an escalatory defensive strategy to deter predation during the vulnerable period between shedding the exuvia and the hardening of the newly formed exoskeleton. In addition, based on sedimentological context (i.e. river- and storm-flood deposits), substrate penetration may have helped to mitigate the relatively high-energy conditions at the sediment-water interface.

In this scenario, the Naranco Formation trace fossils reveal that marginal-marine soft-bodied euarthropods may have been as susceptible to predation as mid-Palaeozoic trilobites living in fully marine environments^8,9,22^, and indicate that infaunal moulting evolved multiple times in different euarthropod lineages as a result of these fundamental selective pressures. This study illustrates the significance of trace fossils for illuminating the evolution of adaptive complex behavioural strategies through deep time and for expanding our knowledge of ecdysis commonly recorded in the form of body fossils^1,23^.

## Materials and Methods

The two stratigraphic sections were measured and their component sedimentary facies described, taken into consideration lithology, physical sedimentary structures, bed boundaries, bed geometry, and fossil content. They were subsequently interpreted in terms of depositional processes and sedimentary environment. Information on the associated trace fossils was integrated with the sedimentary facies analysis. Sixty-five specimens of the moulting trace fossils were collected, and are housed at the Museo del Jurásico de Asturias (MUJA) and Museo de Geología (University of Oviedo) collections. Thirty-three additional specimens were studied in the field at El Tranqueru section (Fig. 1). The horizontal distribution of the specimens preserved on a large bedding plane was mapped (Fig. S3). Fossil material was photographed with a Panasonic Lumix DMC-TZ30 camera fitted with an objective LEICA 1:3.3-6.4/4.3 lens.

## Supplementary information

Supplementary information.
