# Supplementary material for: Trace fossil evidence for infaunal moulting in a Middle Devonian non-trilobite euarthropod
Source: Sci Rep. 2020 Mar 24;10:5316. doi: 10.1038/s41598-020-62019-6 (PMC7093436; doi:10.1038/s41598-020-62019-6)
Supplement: Supplementary file 1 — Supplementary information. [file 41598_2020_62019_MOESM1_ESM.pdf]

Trace fossil evidence for infaunal moulting in a Middle Devonian non-trilobite euarthropod

M. Gabriela Mángano[1*], Javier Ortega-Hernández[2], Laura Piñuela[3], Luis A. Buatois[1], Francisco J. Rodríguez-Tovar[4] & José Carlos García-Ramos[3]

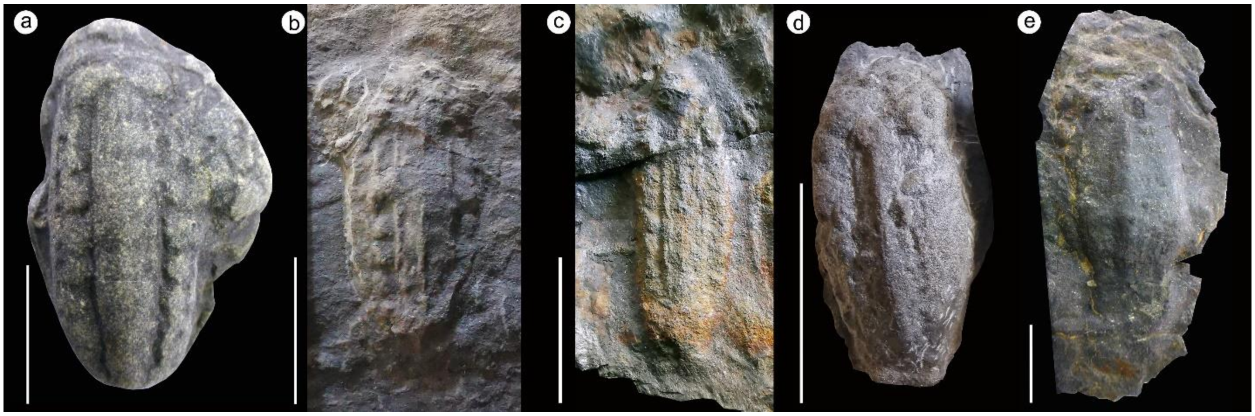

Figure S1. Morphologic variability of morphotype 1 (all specimens with anterior part upwards). **(a)** Highly convex, large specimen showing distinct anterior paired structure, wide axial lobe, clear segmentation and subtle ridge. MUJA-3973. **(b)** Very flat specimen displaying fan-like disturbed anterior area, incipient segmentation and distinct ridge. MUJA-3978. **(c)** Small elongated specimen displaying fan-like, inflated structure. MUJA-4976. **(d-e)** Poorly preserved specimens displaying, moderate convexity, general oval shape and distinct trilobated structure with more convex axial lobe; incipient anterior paired structure. DG-1699 and DG-1694. Scale bars are 2 cm.

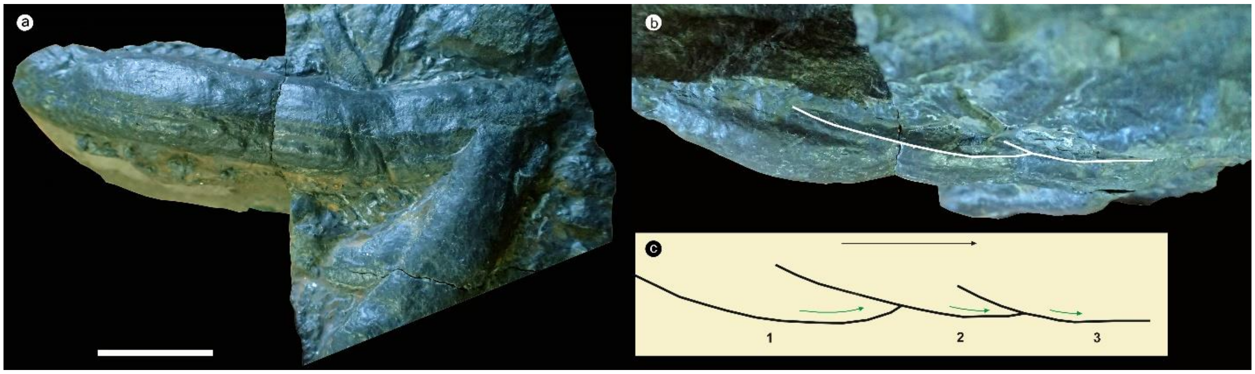

Figure S2. Complex morphotype 2 specimen displaying partially truncated Morphotype 1 specimens. MUJA-4909. **(a)** Bedding-plane view, the sum up of segments 1, 2 and 3 (i.e. partial morphotype 1 specimens) results in an elongated, morphotype 2 structure. **(b)** Cross-sectional view of same specimen revealing different individual components of the structure and cross cutting relationships. **(c)** Interpretative drawing of b highlighting cross-cutting relationships and order of emplacement from 1 to 3. Scale bars are 2 cm.

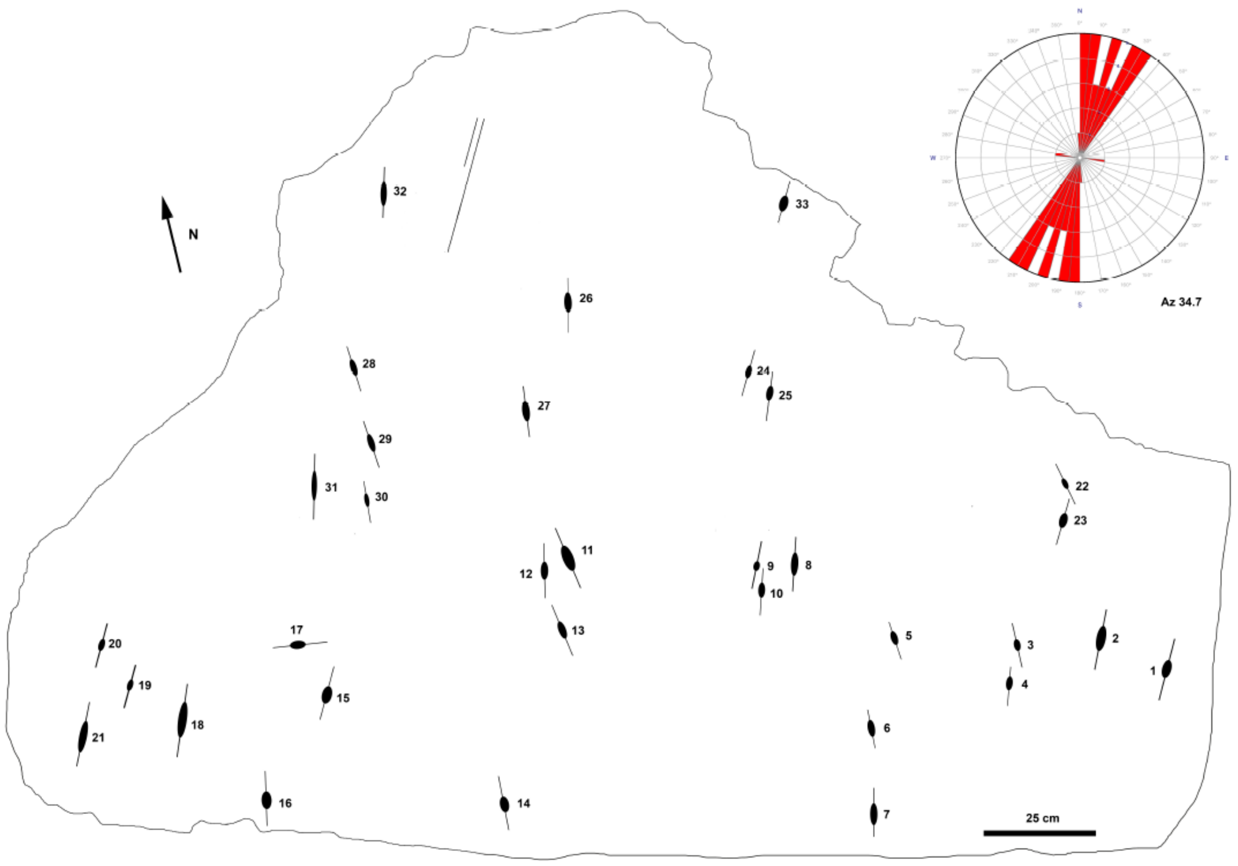

Figure S3. Detailed map of a surface containing a high density of specimens showing a preferred orientation in the El Tranqueru section and accompanying rose diagram with orientation of measured specimens. For location of the surface in the stratigraphic section, see Fig. 2. Map drawn by Laura Piñuela using CorelDraw software version 12.

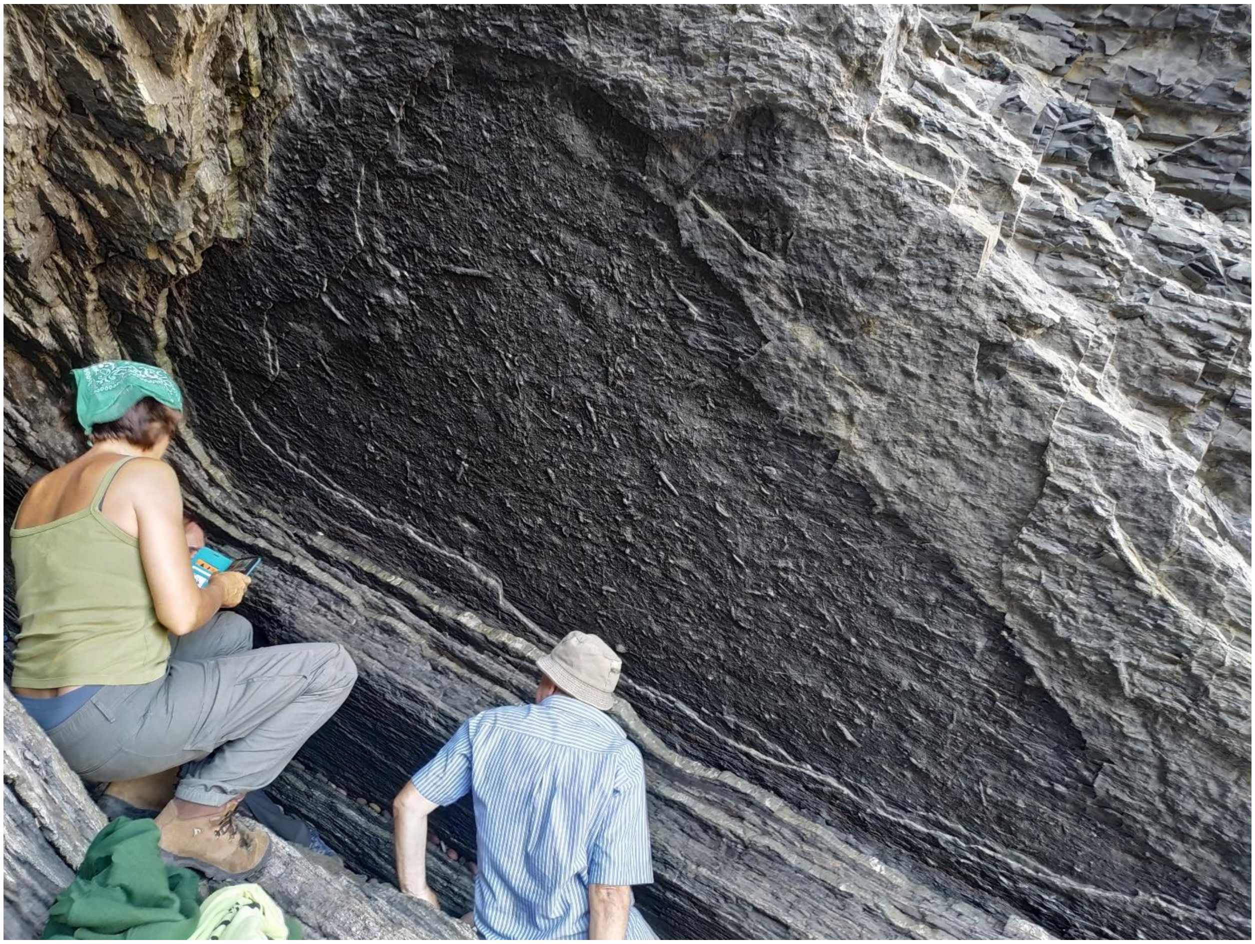

Figure S4. General view of the surface illustrated in Fig. S3.

	Morphotype 1									
	Sample number	Specimen number	Burrow width (cm)	Burrow length (cm)	Segments	Number of segments	Anterior end	Completeness	Locality	Comments
	Jurassic Museum of Asturias collection									
*	MUJA-3973		2,1	5,6		9	APS/F	YES	El Tranqueru	T; smooth, weathered
*	MUJA-3978		1,8	3,8		6	F?	YES	El Tranqueru	R; T; low convexity, poorly preserved
	MUJA-4002		2,0	4,0		6	APS?	NO	Punta La Vaca	R; T; posterior part broken
*	MUJA-4061		1,8	5,3		9	F	YES	Punta La Vaca	R; T; very well-preserved segmentation
*	MUJA-4062		1,7	4,3		5	APS/F	YES	Punta La Vaca	R; T; quality of preservation decreases towards the anterior part
*	MUJA-4064		1,5	4,9	EFF		APS	YES	Punta La Vaca	R; T; highly convex due to presence of another burrow
*	MUJA-4659		1,8	4,8		7?	APS	YES	Punta La Vaca	R; T; low convexity; cross-cut by burrow
*	MUJA-4662		2,0	5,5		9	APS/F	YES	Punta La Vaca	R; T; excellent segmented specimen
*	MUJA-4663		2,0	5,5	EFF		F?	YES	Punta La Vaca	R;T; poorly preserved
*	MUJA-4664	MUJA-4664-1	1,9	5,0		8?	APS/F	YES	Punta La Vaca	R; lateral displacement seen in anterior part
	MUJA-4665	MUJA-4665-1	2,0	3,4		4	APS/F	NO	Punta La Vaca	APS; R; T; cross-cut by burrow
		MUJA-4665-2	2,0	3,5	EFF		NV	NO	Punta La Vaca	R; T; low convexity; cross-cut by burrow
	MUJA-4666		1,2	1,9		5	APS	NO	Punta La Vaca	T; posterior part incomplete
*	MUJA-4813			4,0	EFF		F?	YES	El Tranqueru	T; poorly preserved
*	MUJA-4814	MUJA-4814-3	1,8	4,3	EFF		APS	YES	Punta La Vaca	R; T
	MUJA-4815		1,8	3,5	S		NV	NO	El Tranqueru	T; anterior or posterior part not possible to detect
*	MUJA-4816		1,6	4,2		7	APS	YES	Punta La Vaca	R; T
*	MUJA-4903	MUJA-4903-1	1,1	2,7	EFF		APS?	YES	Punta La Vaca	R; T; low convexity and short
		MUJA-4903-2	1,6	3,1	EFF		NV	NO	Punta La Vaca	R; T; low convexity
	MUJA-4905	MUJA-4905-1	2,0	4,1		6?	NV	NO	Punta La Vaca	R; T; cross-cut by burrow
*		MUJA-4905-2	1,6	5,1	EFF		F	YES	Punta La Vaca	R; T
	MUJA-4906		1,9	4,6		6	APS?	NO	Punta La Vaca	R; T
	MUJA-4908		1,5	3,7		6	NV	NO	Punta La Vaca	R, T; anterior or posterior part not possible to detect
	MUJA-4911		1,7	3,8	EFF		APS	NO	Punta La Vaca	R; T; incomplete; posterior part broken
*	MUJA-4912		1,2	3,7	EFF		APS	YES	Punta La Vaca	R;T
	MUJA-4915		2,1	4,5		6	F	NO	Punta La Vaca	T
	MUJA-4916		1,8	4,1	S		APS	NO	Punta La Vaca	R; T
	MUJA-4917		2,0	4,1		6	NV	NO	Punta La Vaca	R; T; incomplete; broken
	MUJA-4963		2,2	4,3	EFF		NV	NO	El Tranqueru	R; T
	MUJA-4969		1,9	4,5	EFF		APS/F	NO	El Tranqueru	R; T
	MUJA-4970		2,1	2,9	EFF		NV	NO	El Tranqueru	R, very low convexity
	MUJA-4971		2,0	3,7	EFF		NV	NO	El Tranqueru	R; very low convexity
	MUJA-4972		2,2	3,6	EFF	6	NV	NO	El Tranqueru	R; T; low convexity
*	MUJA-4973		2,2	4,6	EFF		APS/F	YES	Punta La Vaca	R; T
*	MUJA-4974		1,9	4,3	EFF		F?	YES	Punta La Vaca	R; T; fan-shaped striations in posterior part
	MUJA-4975	MUJA-4975-2	1,4	2,8	EFF		NV	NO	Punta La Vaca	R; T
*	MUJA-4976		1,2	4,3	EFF		APS	YES	Punta La Vaca	R; T; low convexity
*	MUJA-4978		1,8	4,3		9	APS	YES	Punta La Vaca	R; T
*	MUJA-4980		1,1	2,4	EFF	7?	NV	YES	Punta La Vaca	R; T; one of the smallest specimens
	MUJA-4981		1,8	3,8		7	NV	NO	Punta La Vaca	R; T

	Department of Geology (University of Oviedo) collection									
	DG-1687	DG-1687-3	1,9	4,0	EFF		NV	NO	Punta La Vaca	cross-cut by burrow; slightly displaced
*	DG-1699		1,5	3,4	EFF			YES	Punta La Vaca	R; T; anterior part poorly preserved
*	DG-1701		1,2	3,2	EFF			YES	Punta La Vaca	R; T; anterior part poorly preserved
	DG-1760	DG-1760-1	2,0	4,4	S		APS/F	NO	Punta La Vaca	T; poorly preserved
*		DG-1760-2	2,1	4,4	EFF		APS/F	YES	Punta La Vaca	R; T; poorly preserved
*	DG-1769		1,7	4,5	EFF		APS/F	YES	Punta La Vaca	R; T; poorly preserved
	Samples* in situ *in the El Tranqueru site									
*	1		1,5	3,7	EFF		F	YES		Az 28º; R
*	2		2,1	5,6		4	F	YES		Az 25º; R; at least 4 segments; with radial ridges
*	4		1,3	3,2	EFF		F	YES		Az 20º; R; T
	5		1,6		S			NO		Az 6º; R; very poorly preserved
*	6		1,6	3,4	EFF			YES		Az 4º; R; T
	7		1,8		S			NO		Az 15º; R; T; very poorly preserved; covered with sediment
	8		1,6		EFF			NO		Az 17º; cross-cut by burrow
	12		1,4	2,3	EFF			NO		Az 13º; R; T; very flat
	13									Az 2º; R; T; very poorly preserved; covered with sediment
	15		1,5	2,7	EFF		APS	NO		Az 31º; R; T; cross-cut by burrow
	16		1,9		S		APS	NO		Az 11º; R; T; cross-cut by burrow
*	19		1,1	2,0	S			YES		Az 29º; R; T
	20									Az 30º; R; T
*	22		1,1	2,1	S			YES		Az 179º; R; T
*	23		1,9	4,4		6	APS	YES		Az 30º; R; T; at least 6 segments
*	24		1,3	3,2		4	F	YES		Az 30º; at least 4 segments
	25		1,6	3,4	S			NO		Az 21º; R; T
	26		2,3	4,1	S			NO		Az 13º; covered with mud
	28		1,7	3,6	S			NO		Az 187º; R; T
	29		1,6	3,4	S			NO		Az 6º; broken and weathered
	33		1,8	3,6	EFF/S					Az 30º; partially covered with mud

NV: Not visible
EFF: Effaced
S: Smooth
F: Fan-like
APS: Anterior paired structure
R Ridge
T: Trilobated
* Complete specimen measured

Table S1. Measurements in studied examples of morphotype 1.

Morphotype 2						
Sample number	Specimen number	Burrow width (cm)	Burrow length (cm)	Number of individual longitudinal segment	Locality	Comments
Jurassic Museum of Asturias collection						
MUJA-3870		2,0	6,1	2 (4,5; 1,6)	El Tranqueru	T; EFF
MUJA-4660		1,6	7,8	3 (2,9; 2,7; 2,2)	Punta La Vaca	R; T; at least 7 transverse segments in midldle and posterior longitudinal segment; anteriormost part broken and poorly preserved
MUJA-4661		2,1	6,6	2 (2,8; 3,8)	Punta La Vaca	R; T; at least 6 transverse segments in anterior longitudinal segment and 5 in posterior longitudinal segment; anteriormost part poorly preserved
MUJA-4664	MUJA-4664-2	1,7	4,2	1	Punta La Vaca	R; T; EFF; low convexity
MUJA-4665	MUJA-4665-3	2,0	6,7	2 (4,5; 2,2)	Punta La Vaca	EFF; anterior part poorly preserved
MUJA-4814	MUJA-4814-1	1,8	7,0	2 (4,7; 2,3)	Punta La Vaca	R; T; at least 7 transverse segments in posterior longitudinal segment
	MUJA-4814-2	1,4	5,2	2 (2,9; 2,3)	Punta La Vaca	R; T; at least 5 transverse segments only visible laterally
MUJA-4902		1,5	6,6	1	Punta La Vaca	R; T; S
MUJA-4903	MUJA-4903-3	1,6	11,0	4 (4,5; 1,3; 3,5; 1,7)	Punta La Vaca	R; T; at least 7 transverse segments in anterior longitudinal segment and 8 in another
MUJA-4904		1,4	7,3	4 (2,0; 1,7; 0,9; 2,7)	Punta La Vaca	R; T; EFF; APS
MUJA-4907		2,0	5,7	2 (3,7; 2,0)	Punta La Vaca	R; T; EFF; anteriormost part poorly preserved
MUJA-4909		1,7	10,8	3 (5,5; 2,1; 3,2)	Punta La Vaca	R; T; EFF
MUJA-4910		2,1	7,4	2(6,0: 1,4)	Punta La Vaca	R; T; 8 segments only visible laterally
MUJA-4913		2,4	5,4	1	Punta La Vaca	R; T; APS; subtle lateral displacement
MUJA-4914		1,6	3,7	1	Punta La Vaca	R; T; EFF; anterior and posterior parts cannot be recognized
MUJA-4962		1,7	3,3	2 (2,5; 0,8)	El Tranqueru	R; T; at least five transversal segments on longer longitudinal segment
MUJA-4975	MUJA-4975-1	1,5	4,0	2 (3,2; 0,8)	Punta La Vaca	R; T; EFF
MUJA-4977		1,7	5,9	3 (2,9; 1,7; 1,1)	Punta La Vaca	R; T; EFF
MUJA-4979		1,7	5,1	1	Punta La Vaca	R; T; EFF; APS well developed
Samples in situ on the El Tranqueru site						
3		1,4		1		Az 1º; R; T; S
9				1		Az 26º; R; S; covered with mud (laterally)
10				1		Az 19º; R; S; covered with mud (laterally)
11		1,8	6,7	1		Az 1º; S
14		1,4	6,5	1		Az 4º; R; T; S
17		1,6	11,5	3 (3,1; 4,0; 4,5)		Az 98º; T; EFF
18		1,6	8,5	2 (3,7; 4,8)		Az 22º; T ; EFF
21		1,5	7,9	2 (2,8; 5,1)		Az 25º; R; T; S; broken specimen
27		1,5	4,7	1		Az 7º; R; T; S
30			6,1			Az 5º; R; covered with mud (laterally)
31		0,7	3,1	1		Az 15º; R; S
32		1,3	5,2	2 (2,8; 2,4)		Az 16º; R; S

NV: Not visible
EFF: Effaced
S: Smooth
F: Fan-like
APS: Anterior paired structure
R: Ridge
T: Trilobated
1 segment tubular-elongated with no visible segments
2-3 segments formed by morphotype 1 specimens

Table S2. Measurements in studied examples of morphotype 2.

Morphotype 1												
	All specimens (collected + in the field)		Best-preserved specimens (collected + in the field)		All specimens (collected)		Best-preserved specimens (collected)		All specimens (in the field)		Best-preserved specimens (in the field)	
	Burow width	Burrow length	Burow width	Burrow length	Burow width	Burrow length	Burow width	Burrow length	Burow width	Burrow length	Burow width	Burrow length
n	64	61	31	32	45	46	23	24	19	15	8	8
Average	1.7	3.9	1.6	4.1	1.8	4.1	1.7	4.3	1.6	3.4	1.5	3.5
Standard deviation	0.313	0.871	0.340	0.979	0.306	0.797	0.326	0.825	0.303	0.892	0.341	1.098
Max	2.3	5.6	2.2	5.6	2.2	5.6	2.2	5.6	2.3	5.6	2.1	5.6
Min	1.1	1.9	1.1	2	1.1	1.9	1.1	2.4	1.1	2	1.1	2
Median	1.8	4	1.7	4.3	1.8	4.1	1.8	4.3	1.6	3.4	1.4	3.3

Morphotype 2						
	All specimens (collected + in the field)		All specimens (collected)		All specimens (in the field)	
	Burrow width	Burrow length	Burrow width	Burrow length	Burrow width	Burrow length
n	28	28	19	19	9	9
Average	1.7	6.4	1.8	6.3	1.4	6.7
Standard deviation	0.315	2.116	0.262	2.015	0.290	2.293
Max	2.4	11.5	2.4	11	1.8	11.5
Min	0.7	3.1	1.4	3.3	0.7	3.1
Median	1.6	6.3	1.7	6.1	1.5	6.5

Table S3. Summary of statistical parameters.
